# The Curious Acoustic Behavior of Estuarine Snapping Shrimp: Temporal Patterns of Snapping Shrimp Sound in Sub-Tidal Oyster Reef Habitat

**DOI:** 10.1371/journal.pone.0143691

**Published:** 2016-01-13

**Authors:** DelWayne R. Bohnenstiehl, Ashlee Lillis, David B. Eggleston

**Affiliations:** 1 Department of Marine, Earth & Atmospheric Science, North Carolina State University, 2800 Faucette Dr., Raleigh, North Carolina, 27695, United State of America; 2 Center for Marine Sciences and Technology, North Carolina State University, Morehead City, North Carolina, 28557, United States of America; University of Auckland, NEW ZEALAND

## Abstract

Ocean soundscapes convey important sensory information to marine life. Like many mid-to-low latitude coastal areas worldwide, the high-frequency (>1.5 kHz) soundscape of oyster reef habitat within the West Bay Marine Reserve (36°N, 76°W) is dominated by the impulsive, short-duration signals generated by snapping shrimp. Between June 2011 and July 2012, a single hydrophone deployed within West Bay was programmed to record 60 or 30 seconds of acoustic data every 15 or 30 minutes. Envelope correlation and amplitude information were then used to count shrimp snaps within these recordings. The observed snap rates vary from 1500–2000 snaps per minute during summer to <100 snaps per minute during winter. Sound pressure levels are positively correlated with snap rate (r = 0.71–0.92) and vary seasonally by ~15 decibels in the 1.5–20 kHz range. Snap rates are positively correlated with water temperatures (r = 0.81–0.93), as well as potentially influenced by climate-driven changes in water quality. Light availability modulates snap rate on diurnal time scales, with most days exhibiting a significant preference for either nighttime or daytime snapping, and many showing additional crepuscular increases. During mid-summer, the number of snaps occurring at night is 5–10% more than predicted by a random model; however, this pattern is reversed between August and April, with an excess of up to 25% more snaps recorded during the day in the mid-winter. Diurnal variability in sound pressure levels is largest in the mid-winter, when the overall rate of snapping is at its lowest, and the percentage difference between daytime and nighttime activity is at its highest. This work highlights our lack of knowledge regarding the ecology and acoustic behavior of one of the most dominant soniforous invertebrate species in coastal systems. It also underscores the necessity of long-duration, high-temporal-resolution sampling in efforts to understand the bioacoustics of animal behaviors and associated changes within the marine soundscape.

## Introduction

The combination of physical and biological sounds that form the ambient acoustic environment is increasingly recognized as a fundamental element of habitats [1]. Marine soundscapes are known to influence key ecological processes such as reproduction, larval recruitment and trophic interactions [2–4], and because soundscape properties relate to certain biological and physical properties of an environment (e.g. species abundance, wave action), there is increasing interest in the use of passive acoustic monitoring as a non-destructive habitat assessment tool (e.g., [5–7]). Although the sounds produced by snapping shrimp are often acknowledged as a key component of the acoustic environment in many ecologically and economically important coastal habitats [8–11], the ecology and acoustic behavior of snapping shrimp are poorly understood. Here we examine the acoustic behavior of snapping shrimp within a sub-tidal oyster-reef habitat during a period of approximately one year using high temporal-resolution sampling (2–4 recordings per hour). These data reveal previously unrecognized snapping patterns that strongly influence the local soundscape at diurnal-to-seasonal time scales.

### Snapping Shrimp

Snapping shrimp (Family Alpheidae) are abundant cryptic crevice-dwelling organisms with habitats that include coral and oyster reefs, rocky substrates, and sponge cavities [12]. They are found typically at depths less than a few tens of meters and have an approximate geographic range of ±40° latitude. Although adult snapping shrimp are only a few centimeters in length, these animals possess a claw that can grow to be half the size of their entire body. When this claw is closed rapidly, a cavitation bubble forms and collapses to produce a loud snap sound [13].

There are hundreds of species of snapping shrimp comprising more than two-dozen genera; however, only those species that belong to the genus *Alpheus* and *Synalpheus* are thought to produce vigorous snapping sounds [14]. Their snaps are extremely broadband with energy extending from a few hundred hertz to frequencies of above 200 kHz [15,16]. The amplitudes of snaps generated by adult shrimp have been measured in excess of 190 dB 1 μPa @ 1 m [17–19], making these transient signals among the loudest bioacoustic sounds in the ocean. Large aggregations of snapping shrimp can produce a sustained and distinctive crackling sound that is a major source of biological noise in coastal areas [14,20–22]. It is often the dominant noise source at frequencies above a few kilohertz and may interfere with the use of some systems used for costal surveying and security (e.g., [23,24]).

Snaps are most well studied as signals in conspecific territorial interactions, but also may function to stun prey or deter predators [25,26]. Ant-like eusociality [27,28], aggression [29], and intra- and inter-specific agonistic signaling [30] behaviors have been documented for some species. Strong diurnal and crepuscular patterns in the acoustic activity of snapping shrimp have been reported by several studies; however, the dominance of daytime vs. nighttime snapping appears to vary between habitats and/or species (e.g., [8,21,31]), and variation in temporal patterns is often difficult to interpret due to the use of short-term or synoptic measurements. There also is growing evidence that snapping shrimp sound production may be modulated by abiotic factors, such as water temperature and dissolved oxygen concentration [32,33].

### West Bay Oyster Reserve, Pamlico Sound NC

**Fig 1:**
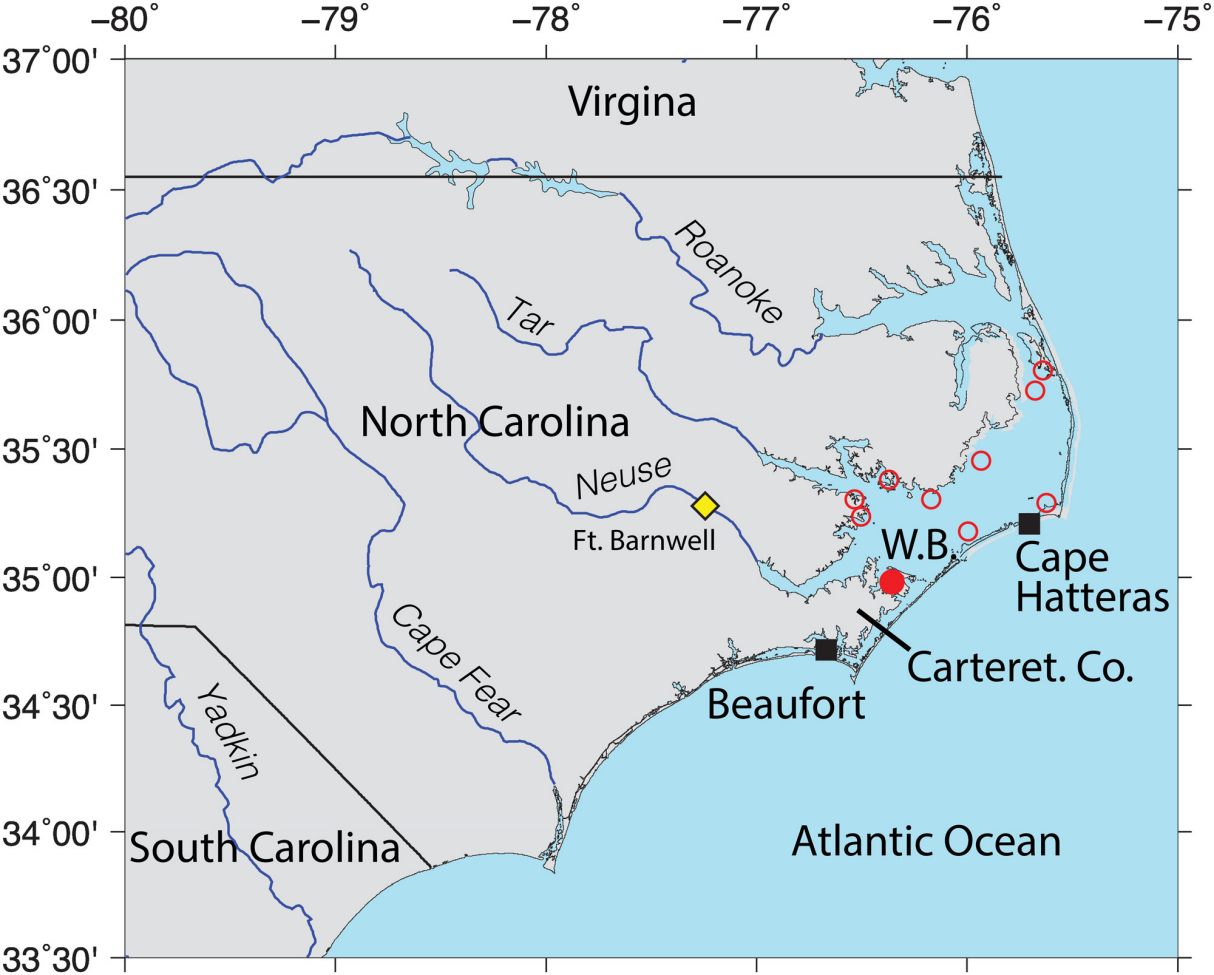
Location Map. Oyster reserve sites in Pamlico Sound, North Carolina, USA (open red circles), including the West Bay Marine Reserve (solid red circle) that was acoustically monitored semi-continuously over the period between 18 June 2011 and 06 July 2012. Water temperature and atmospheric data are available from long-term NOAA monitoring sites in Beaufort and Cape Hatteras NC (black squares). Neuse River discharge data were collected near Fort Barnwell, NC (yellow triangle).

Pamlico Sound is a vast, lagoonal-type estuary located in the southeastern United States within the state of North Carolina (Fig 1). The shallow waters of Pamlico Sound are separated from the Atlantic Ocean by a group of barrier islands and contain a variety of productive nursery and adult habitats for numerous finfish and invertebrate species [34–36].

In 1996, to aid in the recovery of oyster (*Crassostrea virginica*) populations within Pamlico Sound, the North Carolina Division of Marine Fisheries began to create sub-tidal oyster reserves, in which oyster harvesting and the use of bottom disturbing fishing gear are prohibited. Oyster reserves have been constructed throughout Pamlico Sound and range in size from 0.03 to 0.19 km^2^, with inter-reserve distances of 20 to 105 km (Fig 1). Within reserve boundaries, hard materials (e.g. limestone marl, oyster shells) were deployed to provide larval settlement substrate for the development of oyster reefs. Oyster reserves now harbor productive reef communities, including high densities of oysters and numerous resident finfish and invertebrates [36–38]. Oyster reefs within reserves are typically the only three-dimensional substrate on an otherwise relatively featureless bottom of sand, silt or mud.

Acoustic sampling across the estuary has revealed spatially distinct soundscapes [11,39]. Compared to the surrounding off-reef, soft-bottom habitats, oyster reefs in Pamlico Sound exhibit greater acoustic diversity and are characterized by significantly higher sound pressure levels in the snapping-shrimp-dominated frequency band, above ~1.5–2.0 kHz. Field and laboratory-based settlement experiments indicate that the rate of oyster larval settlement increases significantly when larvae are exposed to the oyster reef soundscape, as opposed to the sounds of the ambient off-reef acoustic environment [40,41]. These results are in keeping with a growing body of work that demonstrates the importance of habitat specific soundscapes as a settlement cue in the life cycles of various marine organisms (e.g., [2,40,42–44]).

Given the important contribution of snapping shrimp noise to the soundscape of oyster reefs and other mid-to-low latitude marine habitats, this study investigates the acoustic activity of snapping shrimp within the West Bay Marine Reserve over a period of approximately one year. West Bay is the southern-most oyster reef site within the Pamlico Sound reserve system (Fig 1). The bay and surrounding shorelines are rimmed by undeveloped marshlands that result in a low density of commercial and recreational boat traffic.

Snapping shrimp have not been censused within the West Bay Marine Reserve; however, previous work suggests that *Alpheus heterochaelis* is the dominant local species [26]. In intertidal oyster reefs sampled in the region, they are generally found under large shell clumps partially embedded in the muddy substrate [45]. *A*. *angulosus* also is present within the region, representing 5–25% of the specimens collected nearby in Beaufort NC. They are described mainly in association with areas of loose oyster shell over sand bottom [45].

## Materials and Methods

### Field Recordings and Data

Beginning in June of 2011, a long-term effort was initiated to record ambient underwater sound within the West Bay Marine Reserve (Fig 1). This was accomplished using a low-power DSG acoustic recorder (Loggerhead Instruments, Sarasota, FL, USA) equipped with an off-the-shelf HTI-96 hydrophone with a sensitivity of -185.8 dBV/μPa and flat frequency response between ~0.1 and 30 kHz (High-Tech Inc., Gulfport Mississippi). The instrument was powered by a set of standard alkaline D-cell batteries. The data were digitized using a 16-bit resolution and written to a standard solid-state SD memory card. All components are housed in a 0.65 m long, 11.5 cm diameter PVC pressure case.

The instrument was deployed near the center of the West Bay Marine Reserve at a depth of ~3 m below MLLW and on an area of flat lying seabed ~10 m from surrounding oyster reef habitat. The pressure case was mounted vertically using a concrete anchor to position the hydrophone element ~1 m off the seabed. The tidal range within West Bay is small, typically <0.75 m.

A total of eight deployments were carried out using a single DSG recorder between 18 June 2011 and 06 July 2012 (Table 1). The resulting time series includes data collected on a total of 321 days over this 384-day monitoring period, which represents an 83% data return rate. Depending on the planned length of the deployment, the instrument was programmed to record for a 60 sec duration at 15-minute intervals, or for a duration of 30 sec at 30-minute intervals (Table 1). A 50-kHz sampling rate was used for all deployments. The usable bandwidth of the data is taken to be ~0.1–20.0 kHz, as the DSG applies an anti-alias filter prior to A/D conversion. In total, 27,565 separate recordings were made as part of this monitoring initiative.

**Table 1:** Summary of hydrophone deployments at West Bay Marine Reserve.

	Deployment mm/dd/yy	Recovery mm/dd/yy	Deployment Length	Recording Interval	Recording Duration
1	06/18/11	07/01/11	14 d	15 min	60 sec
2	07/21/11	08/05/11	15 d	15 min	60 sec
3	08/10/11	08/18/11	08 d	15 min	60 sec
4	09/05/11	10/02/11	27 d	15 min	60 sec
5	10/07/11	12/04/11	58 d	30 min	30 sec
6	12/04/11	03/06/12	93 d	30 min	30 sec
7	03/06/12	04/30/12	55 d	15 min	60 sec
8	05/12/12	07/06/12	55 d	15 min	60 sec

The average acoustic variation within each recording is characterized using its root-mean-square (rms) value, and the amplitudes of individual transient snaps are expressed in terms of their peak-to-peak amplitude. Since it is not possible to locate snaps using a single sensor, acoustic source levels are not calculated as part of this study. Previous work by Lillis et al. [39] showed that high-frequency sound levels decay to background levels within ~400 m of the reserve’s boundary. These recordings therefore integrate snaps occurring throughout much of the reserve, although the shallow reef bathymetry likely obstructs propagation from certain azimuths and more distant sources will obviously be received with less amplitude.

To examine potential abiotic variables influencing shrimp snaps, oceanographic and atmospheric data are available from two NOAA monitoring sites within the south-central Pamlico Sound; one is located along the southern margin of the Carteret County peninsula near Beaufort, NC at a distance of ~40 km from West Bay (Station ID: 8656483); and the other is located 60 km to the northeast along the barrier island near the Cape Hatteras inlet (Station ID: 8654467). At Beaufort, water temperatures are measured at a height of 4.4 m below MLLW; at Cape Hatteras they are measured at a height of 1.4 m below MLLW. Both stations also record atmospheric pressure, wind data, and air temperatures. All of these parameters are logged at six-minute intervals and subsequently interpolated at the same 15- or 30-minute intervals used to sample the acoustic data. During the 2011–12 monitoring program, atmospheric pressure (r = 0.25) and wind speed (r = 0.05) vary strongly between sites; however, despite the distance between these stations, air (r = 0.81) and water (r = 0.94) temperatures are highly correlated.

### Snap Detection

Most studies examining snap patterns have used a thresholding technique for detection, whereby a snap is identified whenever the amplitude of the pressure record exceeds a threshold value over a short duration window. This threshold is often selected as a multiple of the longer-term, root-mean-squared pressure of the time series. Typically, a high-pass filter is applied to the waveforms to suppress low-frequency anthropogenic, biologic and geoponic ocean noise, and a minimum delay time between detections is required to prevent the detector from triggering multiple times within the signal coda.

**Fig 2:**
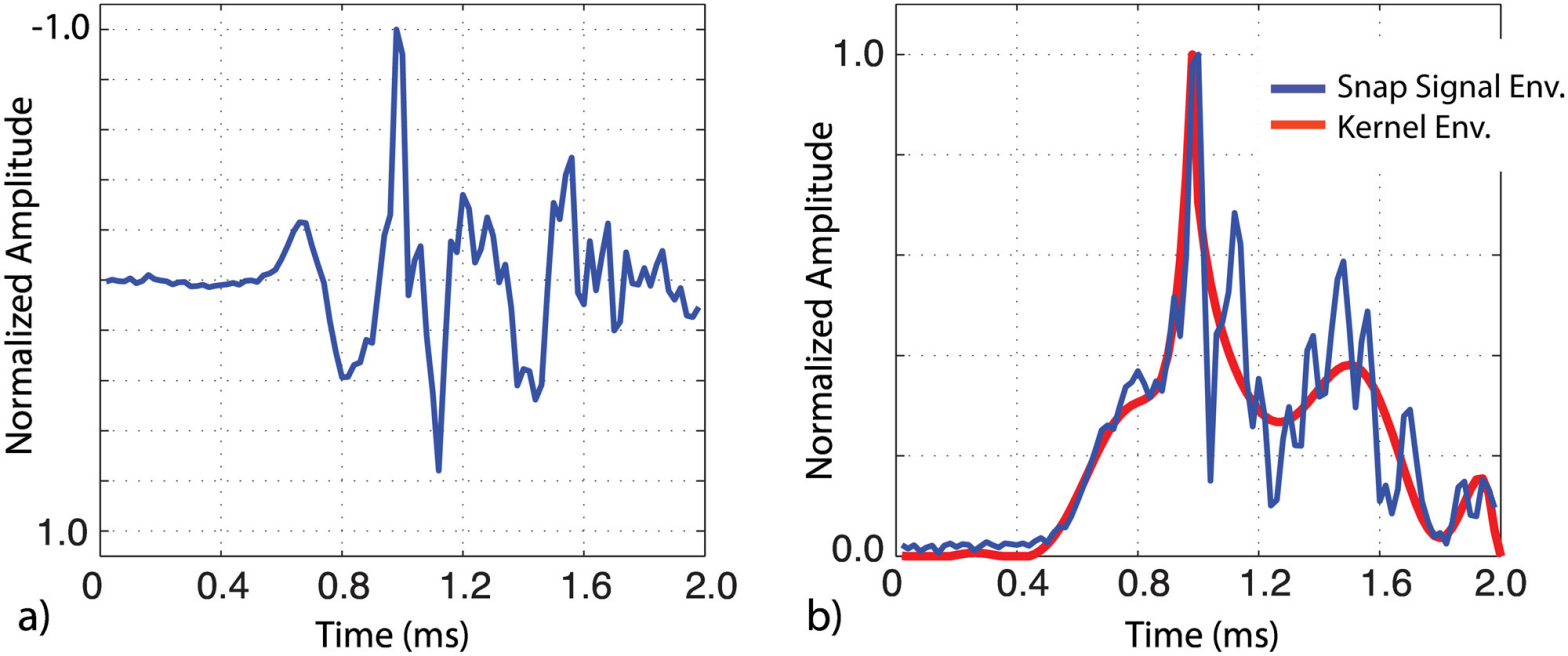
Snap detection using envelope correlation. a) Band-pass filtered (1.5–20 kHz) waveform showing an individual snap. b) The envelope of the snap waveform (blue) and kernel (red). The detection score (correlation) between the signal envelope and kernel is 0.85, sufficient to declare detection.

**Fig 3:**
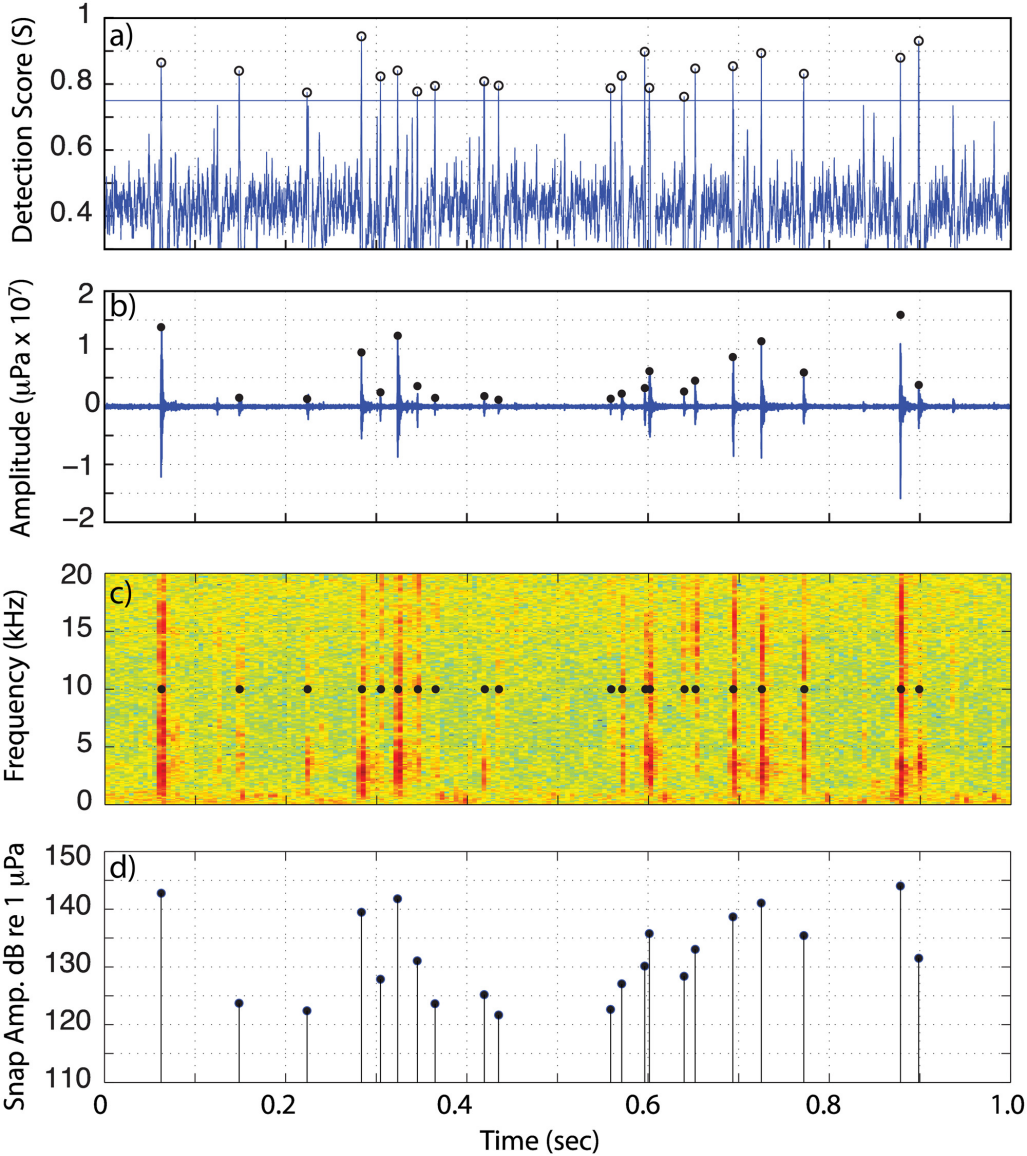
Snap detection example. An example of the detection score procedure applied to the West Bay acoustic data. Circles demote detections with a score S ≥ 0.75 and peak-to-peak amplitude ≥ 120 dB 1 μPa @ 1 m. a) detection score, b) pressure waveform, c) spectrogram, and d) peak-to-peak snap received level.

In this study, we present a novel snap detection algorithm to quantify the acoustic activity of estuarine snapping shrimp. Here, an amplitude threshold is used in combination with a correlation detector that is sensitive to the shape of arriving snap. The correlation score is determined by cross correlating the enveloped time series with a kernel function which represents the smoothed envelope derived from a suite of snaps recorded within West Bay using the DSG system. The enveloped kernel is asymmetric, with a shape that is skewed to the right, and 2.0 ms (100 pts) duration (Fig 2). A zero padding of equal length is applied to the trailing edge of the kernel to suppress multiple detections within the arrival coda. The detection score (S) is expressed in the time domain as the normalized correlation coefficient between enveloped signal and kernel centered at each time step t.

$$S\left(t\right)=\frac{\sum \epsilon \left(t\right)\kappa \left(t\right)}{\sqrt{\sum \epsilon {\left(t\right)}^{2}\kappa {\left(t\right)}^{2}}}$$ (1)

where κ is the kernel time series and ε is a segment of the signal envelope of the same length. The signal envelope is calculated using the Huang Transform applied to a 1.5–20 kHz band-passed waveform. The detection score ranges from 0 to 1, where 1 represents a perfect correlation between the kernel and signal envelopes (Figs 2 and 3).

From these results, the snap rate, expressed as detections per minute, is estimated within each recording. Detections are identified at times when S > 0.75 and the measured peak-to-peak amplitude exceeds a threshold of 120 dB re 1 μPa in the 1.5–20 kHz band (Figs 2 and 3). This amplitude threshold corresponds to the ~90% quantile level of the background noise levels observed throughout the monitoring period. Although smaller amplitude snaps can be detected when the overall noise levels drops during the winter (presumable from sources at a greater range), adopting a lower or seasonally varying threshold does not impact the patterns reported here.

### Time of Day Statistics

To quantify diurnal patterns in shrimp snaps, the number of snaps detected during the day is compared to those detected at night. For each day (00:00–24:00 EST), the percent excess snaps occurring at night (Ω) is calculated as.

$$\Omega =100\frac{N_{o}-N_{e}}{N_{t}}$$ (2)

where *N*_*o*_ is number of snaps observed at night and *N*_*e*_ is the expected number given the fraction of nighttime recordings and the total number of snaps detected daily (*N*_*t*_). Positive values of Ω indicate greater snap activity at night; whereas, negative values of Ω indicate greater activity during daylight hours (cf. [46,47]). Daytime and nighttime periods are defined based on the local sunrise and sunset times for each day.

Using this framework, the probability (*p*) of recording N_o_ or more snaps at night can also be assessed using an exact binomial test:

$$p\left(\geq N_{o},N_{t},\chi \right)=1-\;\sum_{j=0}^{N_{o}-1}\left(\begin{array}{c} N_{t}\\ j \end{array}\right)\chi^{j}{\left(1-\chi \right)}^{N_{t}-j}$$ (3)

where $\left(\begin{array}{c} N_{t}\\ j \end{array}\right)=\frac{N_{t}!}{j!\left(N_{t}-j\right)!}$ and the expected probability (χ) of a randomly occurring snap happening at night is determined by the fraction of nighttime recordings within a given day. Small p-values (e.g., <0.05) indicate significantly more snaps at night than expected; whereas, large p-values (e.g., > 0.95) indicate significantly more snaps during the day than expected.

## Observations

### Seasonal Trends

**Fig 4:**
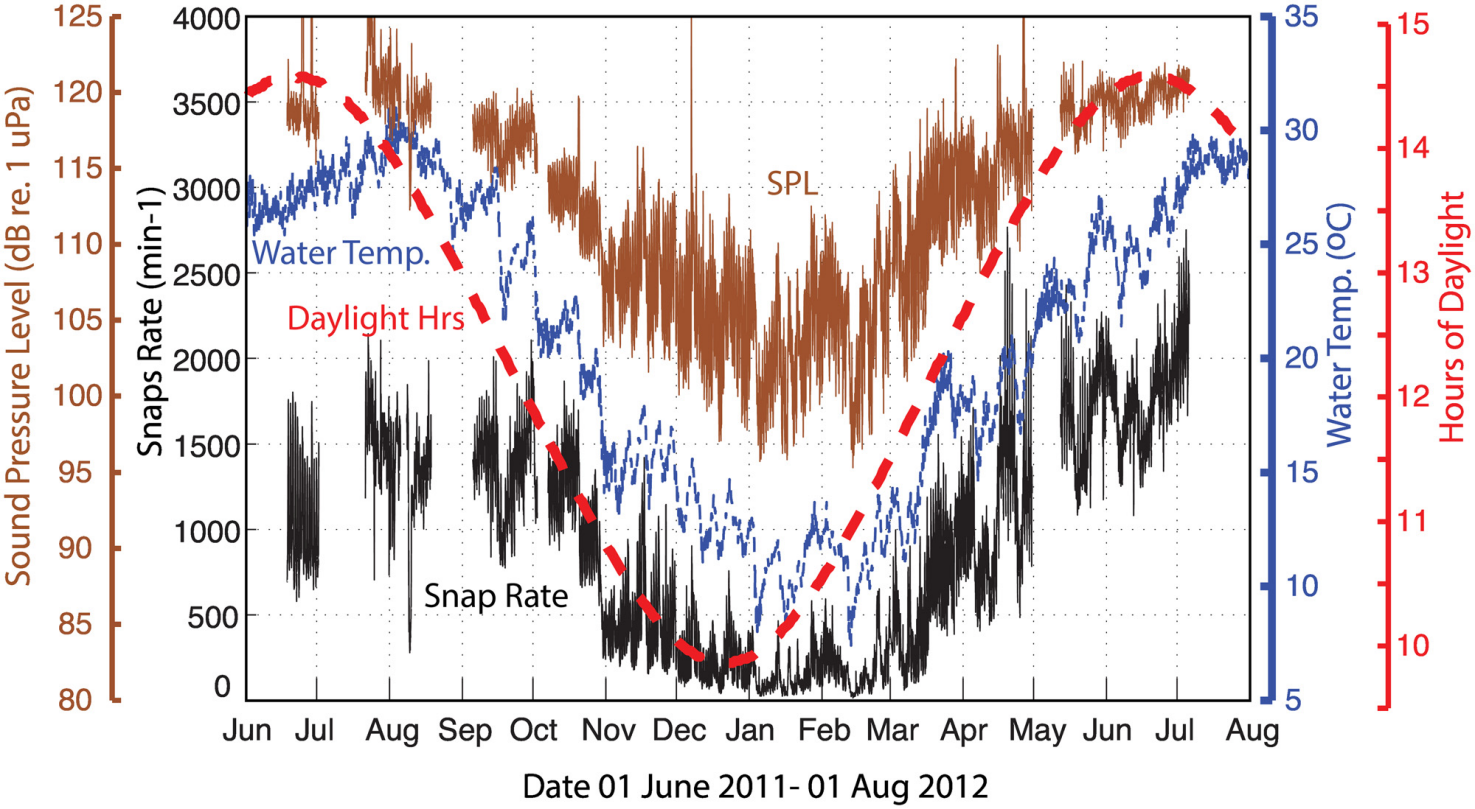
Snap rate vs. time. The solid black line shows number of snaps detected per minute. The dashed blue line shows water temperature in °C from NOAA monitoring site near Beaufort, NC (Fig 1). Dashed red line shows the astronomical length of day in hours. Snap rate and sound pressure level information were smoothed using either a 7 (when recording Interval = 15 minutes) or 3 (when recording Interval = 30 minutes) point filter. Water temperature was interpreted at 15-minute intervals and smoothed using a 7-point filter.

Fig 4 shows the snap rate through the monitoring period of June 2011 through July 2012 (Table 1). Snaps are detected relatively more frequently in the summer versus winter months, reaching an average of ~1500 snaps/min in July of 2011 and ~2000 snaps/min in July 2012, and dropping to a minimum value of less than 100 snaps/min in January. This annual pattern follows in-phase with changes in water temperature and slightly lags seasonal changes in light availability or length of day (Fig 4). Shifts of a few degrees in water temperature, which occur over periods of days to weeks in the spring and fall, can be seen in Fig 4 to be met by an accompanying change in the baseline snapping-rate. This same seasonal trend is also present in the high-frequency (1.5–20 kHz) noise band, with sound pressure levels observed to be on the order of 120 dB re 1 μPa during the summer and 105 dB re 1 μPa during the mid-winter months (Fig 4).

**Fig 5:**
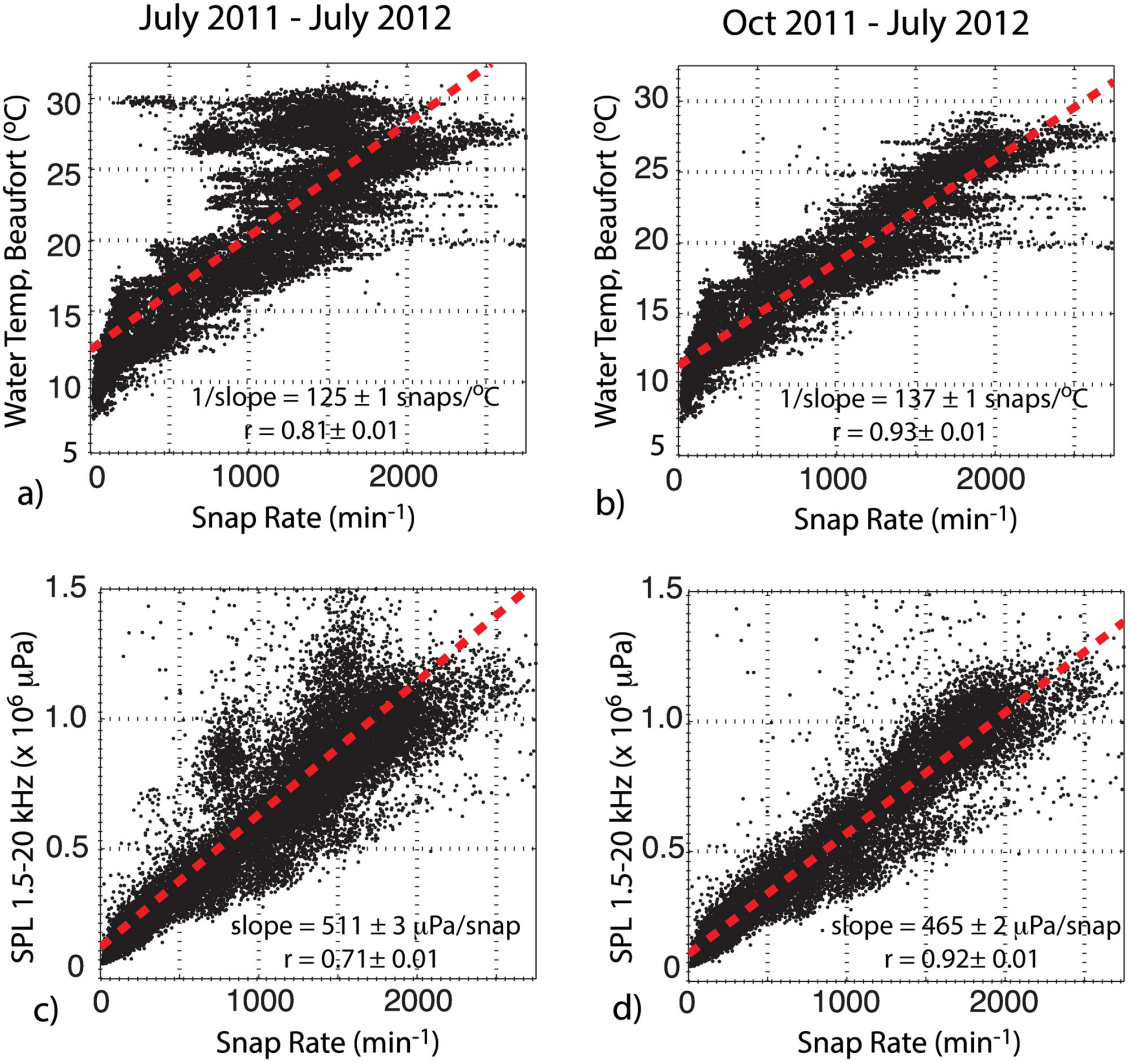
Snap rate, SPL and water temperature correlations. Panels (a) and (b) show the snap rate in West Bay vs. water temperatures measured at station (Beaufort, NC) for the periods July 2011-July 2012 and Oct 2011 –July 2012, respectively. Panels (c) and (d) show snap rate vs. root-mean-square sound pressure levels during these same periods. Red line shows least squares fit to the data. Slopes and correlation coefficients are shown in each panel; uncertainties are estimated using a bootstrap resampling.

Over the ~12 month monitoring window, water temperature and snap rate are linearly correlated at a level of 0.81 ± 0.01 (Fig 5a). Increased scatter in this relationship is observed at high water temperatures, reflecting inter-annual differences between the summer of 2011 and summer of 2012. If the data extending between Oct 2011 and July 2012 (9 months) are considered separately, this correlation increases to a value 0.93 ± 0.01 (Fig 5b). Similarly, snap rate and sound pressure levels display a correlation coefficient of 0.71± 0.01 and 0.92± 0.01 over these respective time windows (Fig 5c and 5d).

### Daily Trends

**Fig 6:**
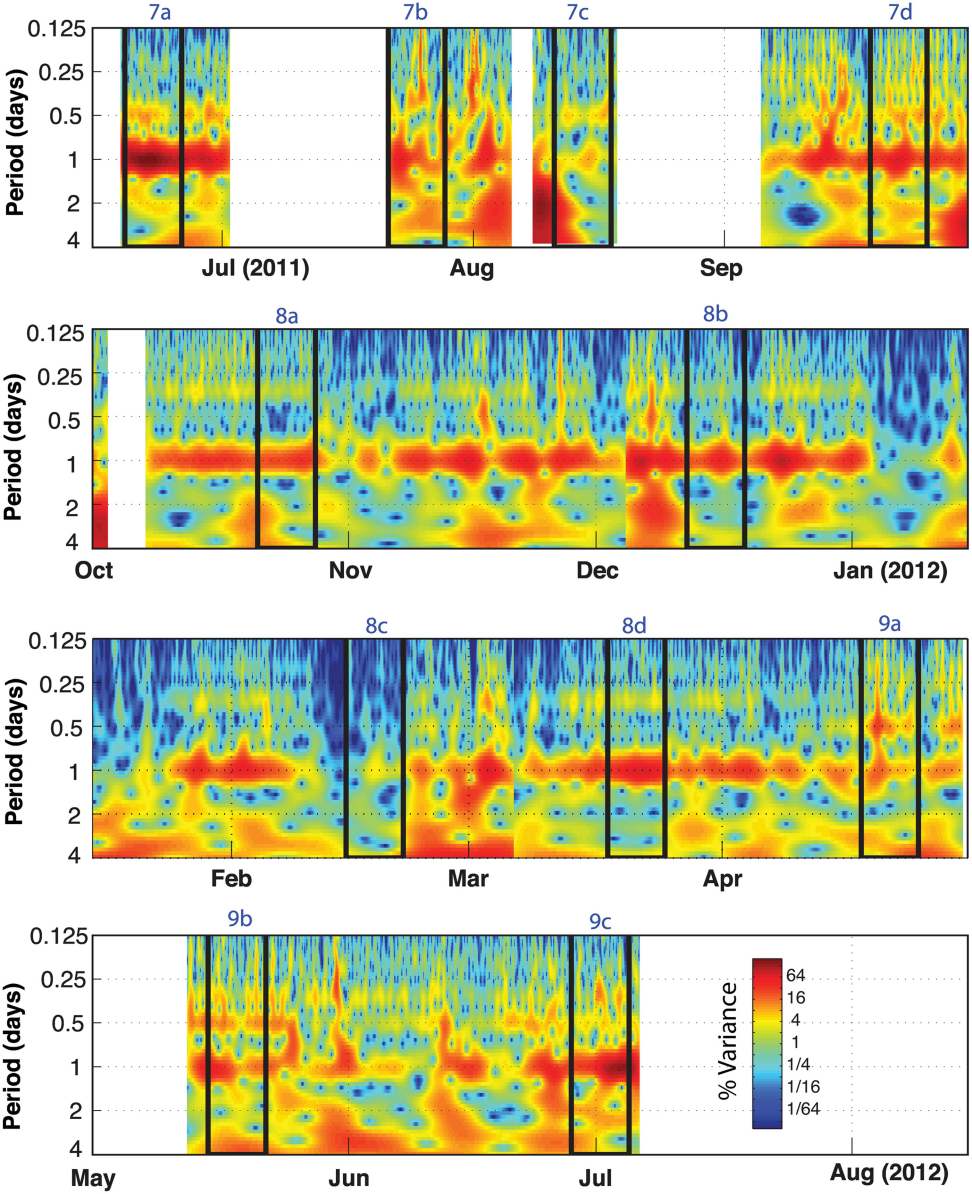
Wavelet analysis. Wavelet scalogram of the snap rate data generated using a Morlet wavelet. The black boxes delineate the 1-week duration time series examples shown in Figs 7–9. Unlike short-time Fourier techniques, the wavelet transform method makes use of variable length analysis windows and can often provide better temporal resolution than fixed-window-length spectral methods. The figure highlights the persistence, and varying strength, of the diurnal periodicity in snap count throughout the monitoring period. The snap rate time series intermittently exhibits additional variance at longer periods (2–4 days), which may be related to variations in water temperature (Fig 4) or water quality.

**Fig 7:**
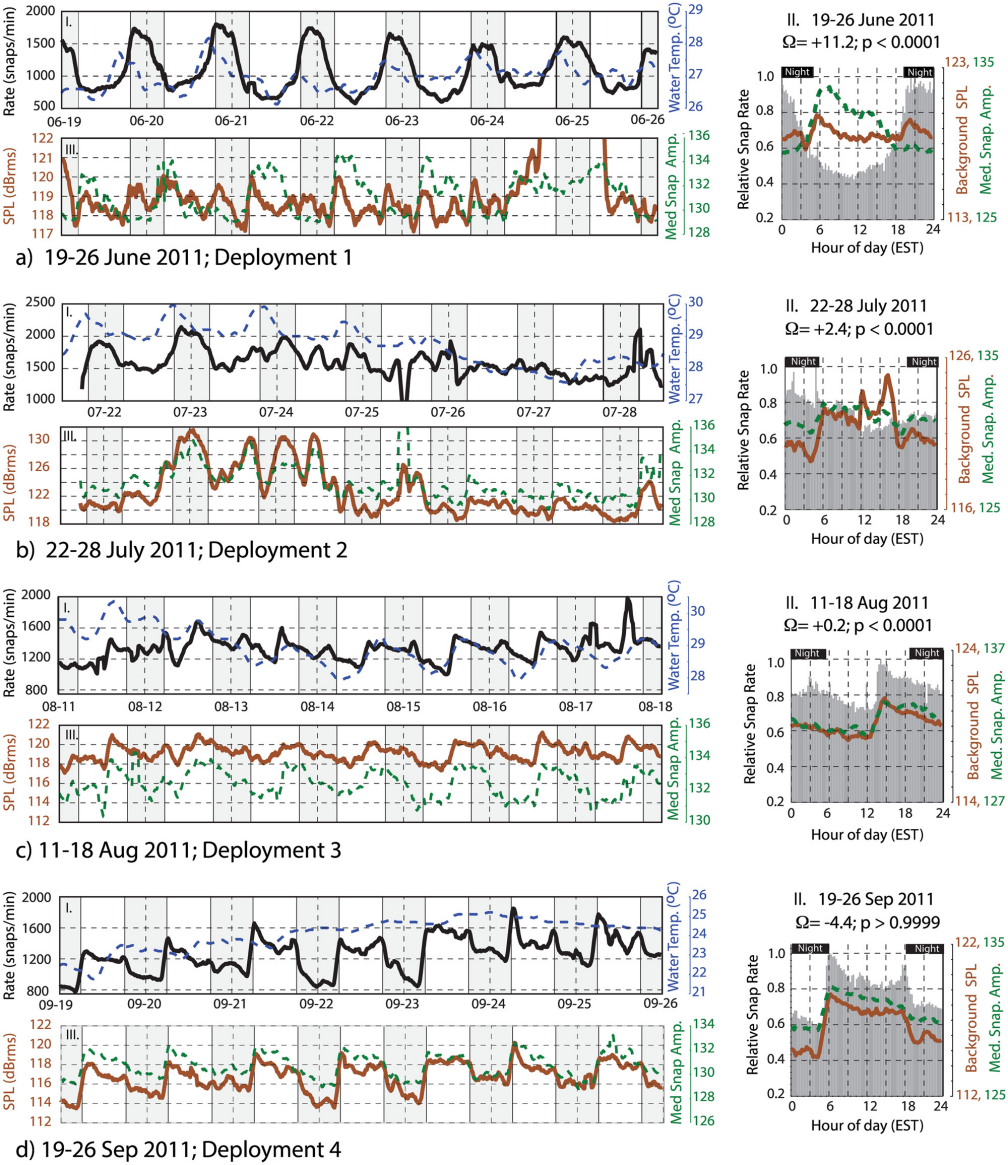
Time series examples June 2011- September 2011. A series of one-week duration data windows are displayed. Date ranges and deployment numbers are shown for each example. Panels I and III show time series examples of snap rate (black), water temperature (dashed blue), 1.5–20 kHz band rms sound pressure level (brown), and median peak-to-peak snap amplitude (dashed green). All pressure data have units of dB re 1 μPa. The vertical scale is optimized for each week’s data to better visualize the patterns in panels I and III. The panels labeled II show weekly histogram plots of the relative number of snaps (black), along with the median sound pressure level (brown) and snap amplitude (dashed green) as a function of time of day. Black horizontal bars define nighttime periods. The vertical scale ranges are fixed for each panel II to represent the relative strength of the diurnal pattern from week-to-week. The percent excess within each of these weekly data windows is labeled. All time series are smoothed using either a 7 (when recording Interval = 15 minutes) or 3 (when recording Interval = 30 minutes) point filter. Time series example continued in Figs 8 and 9.

**Fig 8:**
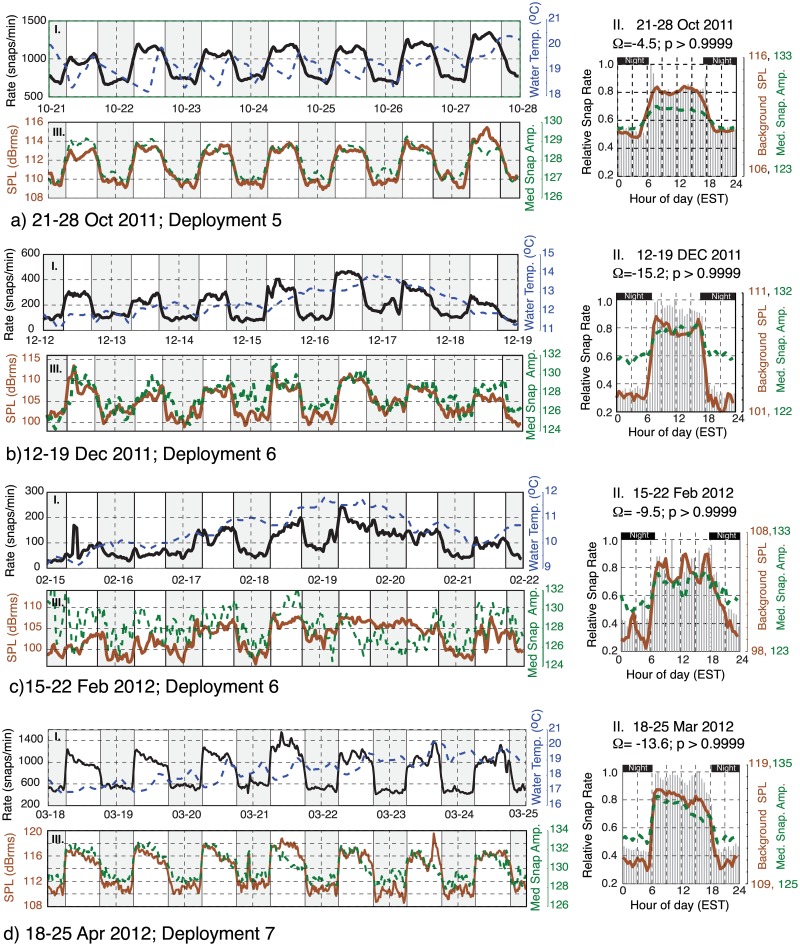
Time series examples October 2011- April 2012. Panels I and III show time series examples of snap rate (black), water temperature (dashed blue), 1.5–20 kHz band rms sound pressure level (brown), and median peak-to-peak snap amplitude (dashed green). The panels labeled II show weekly histogram plots of the relative number of snaps (black), along with the median sound pressure level (brown) and snap amplitude (dashed green) as a function of time of day. See Fig 7 caption for additional information. Time series continued in Fig 9.

**Fig 9:**
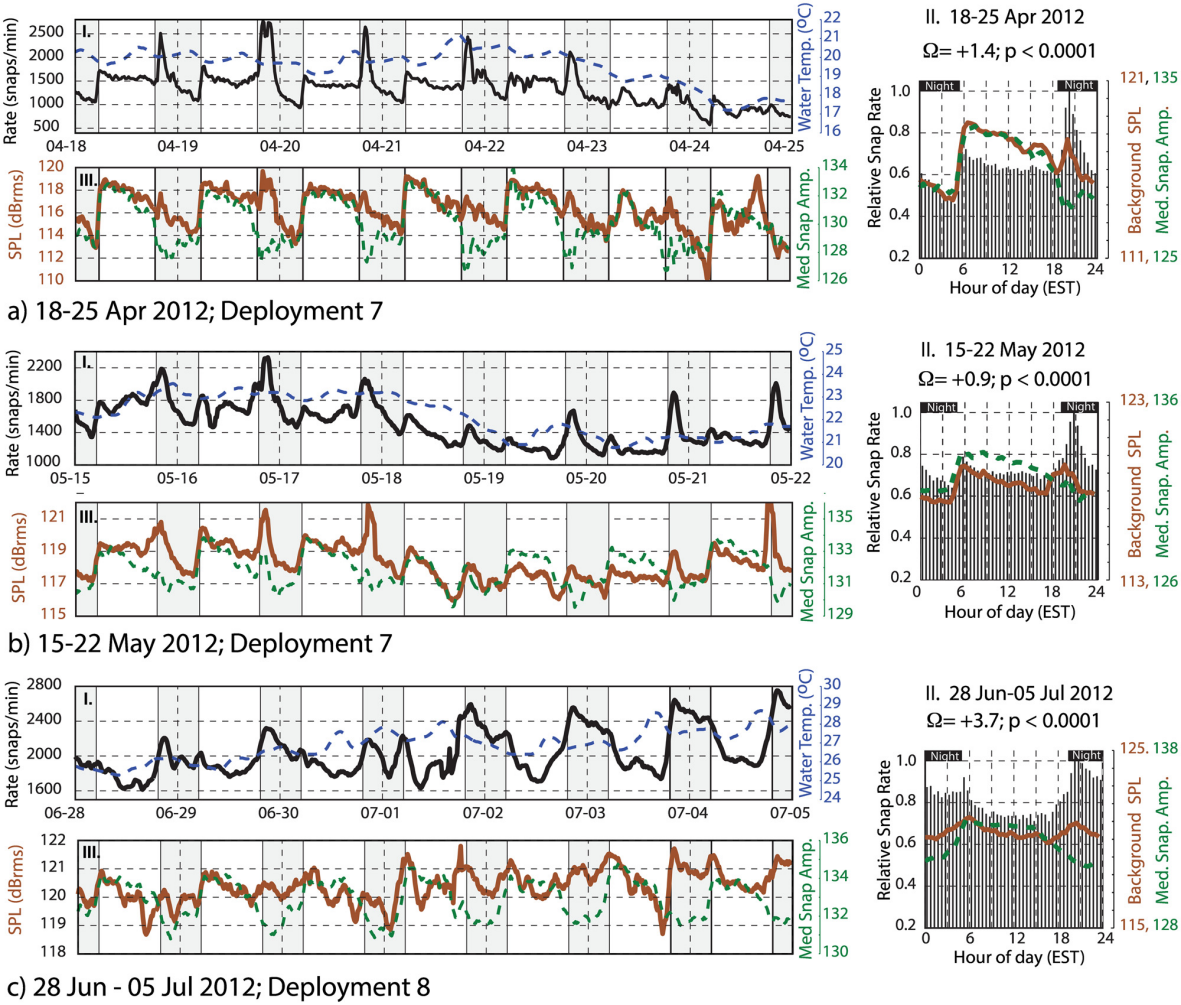
Time series examples April 2012- July 2012. Panels I and III show time series examples of snap rate (black), water temperature (dashed blue), 1.5–20 kHz band rms sound pressure level (brown), and median peak-to-peak snap amplitude (dashed green). The panels labeled II show weekly histogram plots of the relative number of snaps (black), along with the median sound pressure level (brown) and snap amplitude (dashed green) as a function of time of day. See Fig 7 caption for additional information.

A time-frequency representation of the snap rate data is shown in Fig 6 using a Morlet wavelet scalogram. These results reveal the presence of a prominent diurnal (once per day) periodicity in snap counts. This periodicity persists throughout the monitoring period, although it is observed to lose strength intermittently (e.g. Jan 2012). To investigate the timing of these diurnal snap patterns, Figs 7–9 (panel I) display a set of example 1-week duration time series windows that show the snap rate data plotted along with the water temperature records from Beaufort, NC. Figs 7–9 (panel II) show the relative frequency of these snaps with respect to the time of day. These data segments span the eight deployments and were selected to highlight the variable diurnal patterns observed over the course of the year. For reference, the timing of these time- series examples is indicated on the Fig 6 scalogram. Start and end time of each deployment are given in Table 1.

During deployment 1 in June of 2011, the snap rates are elevated during nighttime hours with a strong diurnal pattern (Figs 6, 7a-I and 7a-II). There is a slight asymmetry to the snap rate pattern throughout this time, with the peak in rate observed a few hours after dusk and a minimum observed mid-day (Fig 7a-I and 7a-II). Over daily time scales, snap rate and water temperature do not vary in phase; rather, water temperature, which has a daily range of ~ 1°C at this time, peaks near sunset.

Following a three-week hiatus in monitoring, the first few days of deployment 2 (21–24 July) show the same pattern of nighttime preference for snap activity (Fig 7b-I and 7b-II). A strong diurnal pattern is not observed through the remainder of deployment 2 (ending on 05 Aug) or the entirety of deployment 3 (10–18 Aug). There is, however, an afternoon peak in activity during much of deployment 3, and briefly the snap rate varies in-phase with diurnal water temperature variations, which remain small (~ 1°C range) (Fig 7c-I and 7c-II).

As monitoring resumed in September of 2011 during deployment 4, after an 18-day hiatus, a strong diurnal periodicity is again observed (Fig 6). The time of greatest snap activity, however, is now aligned with the daytime hours (Fig 7d-I and 7d-II). This pattern of predominant daytime activity continues through March of 2012 (deployments 5–7; Fig 8). Water temperature records exhibit little-to-no daily variation throughout the winter of 2011–2012; however, the daily average snap rate does track water temperature at longer (multi-day) time scales (Figs 8b and 8c and 9a–9c).

In mid-April of 2012, near the end of deployment 7, the diurnal snapping pattern shifts again. A narrow daily peak in snap count is observed ~1 hour after sunset, with the rate then decreasing each night. This is followed by a sharp increase in activity at sunrise and a rate that is then maintained at a near constant level throughout the daylight hours (Fig 9a and 9b). The pattern, however, continues to evolve and by late June 2012 a nighttime snapping predominance is again present (deployment 8, Fig 9c-I and 9c-II)—similar to what had been observed when monitoring first began in June of 2011 (Fig 7a-I and 7a-II). Once again, water temperature exhibits little-to-no daily variation; however, the daily average snap rate continues to track water temperature at longer time scales.

**Fig 10:**
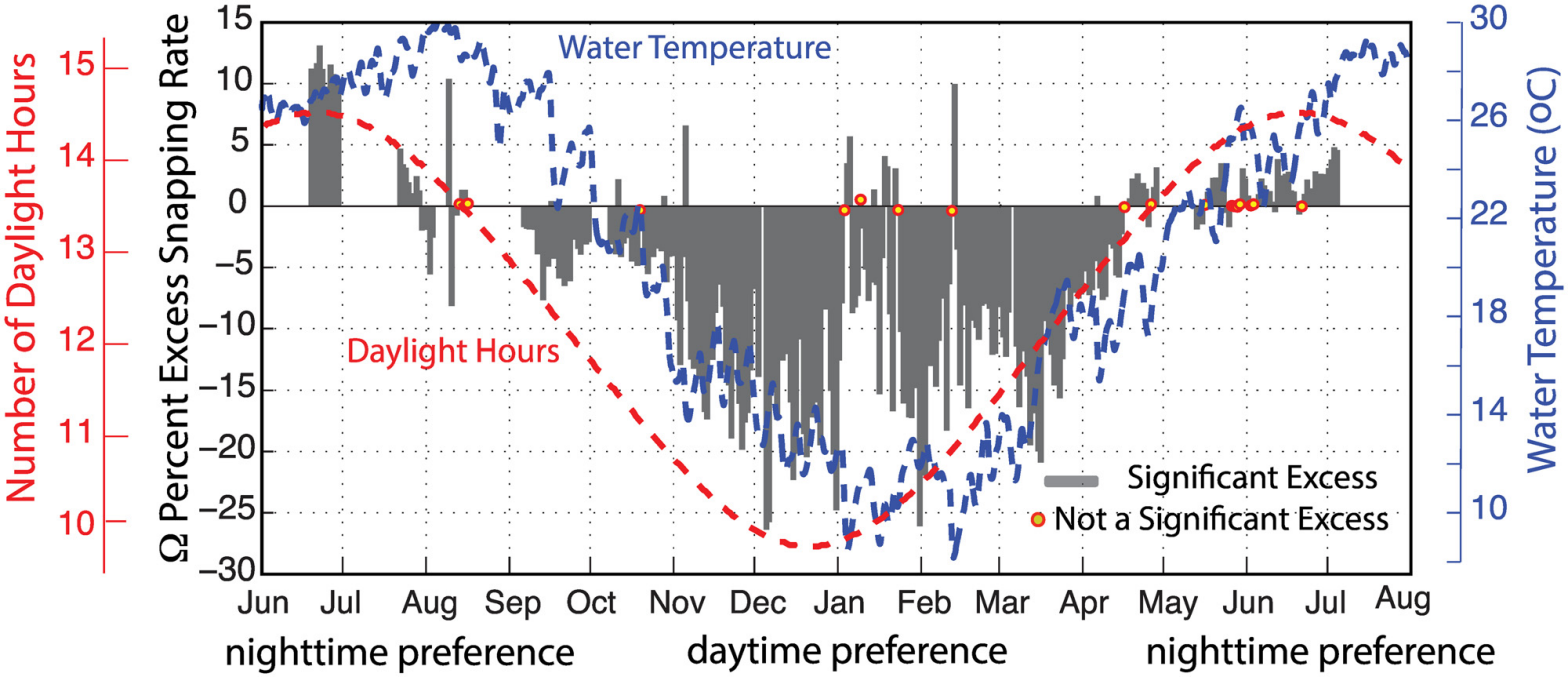
Diurnal snapping pattern. Percent excess snap count throughout the 2011–2012 monitoring period within the West Bay Marine Reserve. Grey bars show the daily percent excess values that are statistically significant (95% confidence); circles denote days when small percent excess values are not significantly different. Positive percent excess indicates a preference for nighttime snapping; whereas, negative values indicate a preference for daytime snapping. The water temperature data from Beaufort NC are shown in blue, and the length of day in hours is shown red.

Fig 10 summarizes the day-night patterns in snap rate using the percent excess calculated for each day. The percent excess peaked in June/July (Ω = 5–12%) and reached a minimum in December (Ω = -25%) near the summer and winter solstices, respectively. The trend in day-night snap count excess is therefore that of an asymmetric sinusoid that tracks the length of day—as opposed to the water temperature, which peaks in late summer. The transition between positive and negative excess values occurred near the beginning of August in 2011 and during mid-April of 2012, with the strength of the day-night pattern weakening near the transition time. A significant excess of either nighttime (*p* <0.05) or daytime (p > 0.95) snapping is observed on 95% of the recording days.

Figs 7–9 (panels *II* and *III*) also show the background SPL (1.5–20 kHz), along with the median amplitude of the detected snaps. During the mid-winter months, when snapping occurs preferentially during the daylight hours and the magnitude of the percent excess is the largest, daytime SPL increases by ~5–6 dB relative to the nightly lows (Fig 8b and 8c). Median snap amplitudes follow a similar pattern, rising by 2–3 dB during the mid-winter days (Fig 8b and 8c).

During the summer months, when the number of snaps occurring at night is in excess (Figs 7a and 9b and 9c), the observed change in sound pressure level between the daytime and nighttime periods is small (< 1 dB) or absent. Median snap amplitudes, however, are still elevated during the daylight hours, relative to the night, and often display a peak just after sunrise (Figs 7a and 9b and 9c).

## Discussion

Broadband acoustic monitoring in the temperate waters of oyster reef habitat within the West Bay Marine Reserve was conducted with high-resolution temporal sampling (15–30 min) over a period of more than one year—revealing complex snapping shrimp acoustic behaviors that have not been documented previously. Although our monitoring program targeted only a single site, the dataset provides a rare opportunity to examine in detail the acoustic behavior of snapping shrimp across multiple temporal scales. To date, only a handful of studies have investigated the acoustic behavior of snapping shrimp with similar temporal resolution and duration (e.g., [31,33]). Much of what is known about the acoustic ecology of these animals is therefore based on short duration field surveys. Furthermore, most temporal analysis has looked exclusively at the pattern of high frequency acoustic energy (e.g., [31,33]), as opposed to the more data-rich snap detection approach implemented in this study.

In tropical regions, where there is little temporal variation in water temperature, snapping rates and high frequency sound levels have not been reported to vary systematically throughout the year [14,25]. In temperate waters, however, measurements do show seasonal patterns. Near coastal South Korea (35° N latitude), for example, Jung et al. [33] reported that under low wind conditions, high frequency snapping-shrimp dominated sound levels vary by as much as ~7 dB (at 20 kHz) as local water temperatures range from 9.9 to 18.4°C. In this study, ambient high-frequency (1.5–20 kHz) sound levels also varied strongly with seasons, ranging by ~15 dB between wintertime low (7°C) and summer time high (30°C) temperatures. In coastal Japan, snap detection results were used by Watanabe et al. [32] to investigate seasonal changes in the acoustic behavior of snapping shrimp. Using approximately 30 two-minute-recordings taken from multiple sites, they suggested a positive exponential relationship between snap rate and water temperature. A simpler linear model, however, is favored in the regression of the much larger West Bay dataset, with water temperature explaining 65–86% of the variance (r = 0.81–0.93) in snap rate. The relationship of snapping activity with water temperature is likely driven by a combination of seasonal changes in the snapping shrimp population size, as well as the direct influence of water temperature on animal activity.

**Fig 11:**
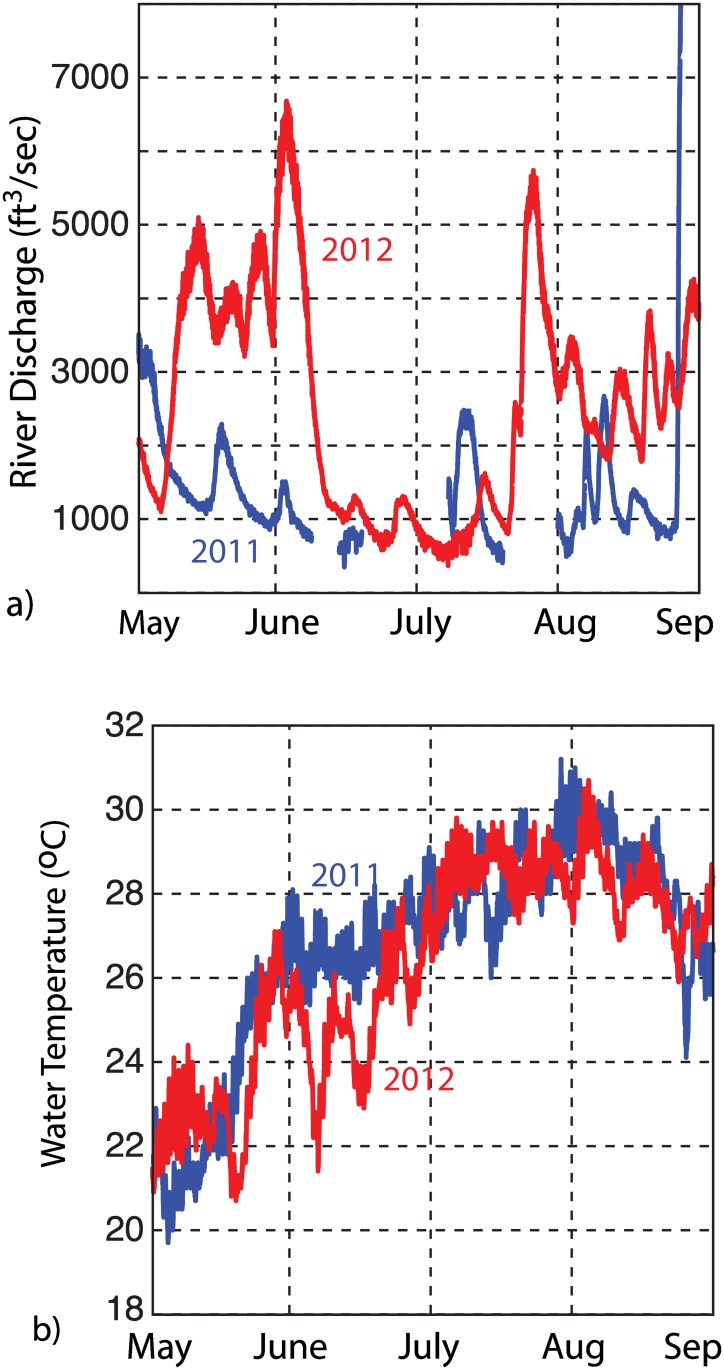
Inter-annual climate variability. Environmental data from Pamlico Sound, NC during the summer of 2011 (blue) and 2012 (red). a) River discharge measured on the Neuse River near Fort Barnwell, NC (35.31°N, 77.30°W). b) Water temperature measured near Beaufort, NC.

Snap rates at West Bay were lower during the summer of 2011 than during the summer of 2012; yet, somewhat anomalously, the background SPL showed only slight differences between the two years (Figs 4 and 5). This could suggest an increase in other biological or anthropogenic sources during the summer of 2011. In the absence of significantly higher SPLs, however, it seems unlikely that the lower snap rates observed in the summer of 2011 were purely an artifact of detection masking. Alternatively, inter-annual changes in snap rate could be driven by changes in water quality or salinity. Near the Japanese coast, for example, snap rate decreased under conditions of low dissolved oxygen (DO) concentrations [32]. While abiotic factors were not monitored locally within the West Bay Reserve, previous work within the adjacent Neuse River section of the estuary suggests that river discharge and temperature are important factors in controlling the quality of surface waters (e.g., [48]). Fig 11 shows discharge from a gauging station near Fort Barnwell, NC (Fig 1), along with water temperature record from Beaufort, during the summers of 2011 and 2012. The comparison indicates extremely low flow conditions throughout the summer of 2011 and water temperatures that are sustained at slightly elevated values, relative to 2012. Climate-driven variability in water quality or salinity in West Bay could therefore contribute to the observed difference in snapping rate during these two summer periods. Future efforts should incorporate *in situ* monitoring of the bottom waters to assess the relationship between abiotic factors and snap rate.

Diurnal and crepuscular patterns of snapping activity, or more commonly high-frequency noise levels, have been reported in a number of soundscape characterization studies (e.g., [8,9]). In the laboratory, *A*. *macellarius* displays diurnal patterns in activity such as burrow construction and foraging, with shrimp in experimental tanks being more active in the morning and late afternoon, and often hidden or resting at midday [49]. Nolan and Salmon [26] found a crepuscular peak and nighttime increase in sound production and locomotor activity by groups of *A*. *heterochaelis* in the laboratory. Although these observations cannot be directly transferred to other shrimp species in the wild, they demonstrate how diurnal patterns in an acoustic activity might be linked to behavioral shifts throughout the day.

Our observations within the temperate, turbid waters of the West Bay Marine Reserve show a shift from preferential nighttime snapping in the summer to preferential daytime snapping in the winter. These shifts occur in August (2011) and April (2012), with the percentage of excess snaps peaking near the summer and winter solstices. The transition between dominant daytime or nighttime snapping and the strength of the diurnal pattern appear to relate to light availability. It is not clear, however, if this transition is driven by changes in the composition (or acoustic activity) of species, or related to behavioral patterns associated with reproduction, resource availability or predator populations.

Numerous short-term measurements (days) in both temperate and tropical waters have suggested a preference for nighttime snapping (e.g. [21,50]), similar to what is observed during the summer time at West Bay, but a transition between daytime and nighttime snapping has not been identified previously in the literature. The high-frequency (2–10 kHz) soundscape data presented in Staaterman et al. [9] (see their Fig 3), however, suggests higher daytime snapping shrimp acoustic activity during certain periods of the year at one coral reef site within their longer-term study sites within the Florida Keys. Moreover, recordings by Lammers et al. [31] in Hawaii have shown both tendencies at nearby sites—with nighttime snapping favored within Kaneohe Bay and daytime snapping favored within Waikiki Conservation District just 25 km away.

In general, there was a tendency to record louder snaps (higher median peak-to-peak amplitudes) during the daylight hours at West Bay. This observation holds throughout the year. Because detection techniques are sensitive to the signal-to-noise ratio, within the winter months such a pattern could simply be an artifact of there being higher background noise (more snaps) during daylight hours. However, the persistence of this pattern during the summer, when there is little diurnal change in the background noise levels, suggests that larger clawed animals, which should produce louder snaps [17], may be more acoustically active during the daylight hours. This could reflect a shift in the relative activity of either different species, different stages of shrimp (e.g. juvenile vs. adult) or potentially male and female shrimp that exhibit claw size dimorphism.

## Conclusions

The rate of snapping shrimp activity within the West Bay Marine Reserve drives the temporal pattern of the high-frequency (>1.5 kHz) soundscape. High temporal resolution and long duration monitoring indicates the snap rate varies linearly with water temperature, dropping by more than an order of magnitude between the summer and winter months. This patterns results in a ~15 dB seasonal change in high-frequency noise levels.

Snapping shrimp also appear to be influenced by light availability, with increased crepuscular activity and significantly elevated snapping rates observed during either the daytime or nighttime on most days. In the mid-summer, there is an excess of 5–10% more snaps occurring at night; whereas in the mid-winter this pattern is reversed, with an excess of up to 25% more snaps recorded during the day. While both daytime and nighttime increases in snap rate have been reported in the literature, this is the first study to show a shift in behavior at a single site. The seasonal shift is particularly intriguing because it is not easily explained by what is known about snapping shrimp behavior and life history patterns. When the snap excess is largest within the mid-winter months, the high-frequency soundscape varies by as much of 5–6 decibels during the course of the day; whereas during the summer, there is little-to-no diurnal change in sound pressure levels.

Marine life interacts with the ocean acoustic environment in many ways, and a range of invertebrate, fish and marine mammal species can detect the broadband, high source-level signals generated by snapping shrimp [51–53]. Understanding the temporal dynamics of snaps therefore informs us broadly about the acoustic ecology of coastal and estuarine environments. The temporal changes in background noise, which are driven by the acoustic behavior of snapping shrimp, may impact the call detection capabilities of some animals (masking) and can be an important consideration in efforts to monitor ecosystem health using passive acoustics [5,7,31]. The performance of active and passive sonar systems can also be affected by the temporal pattern of snapping; this may impact a variety of oceanographic surveying, underwater communications and harbor security applications.

This study highlights the value of long-term high-resolution acoustic monitoring and signal detection approaches in soundscape research. It demonstrates that short-term synoptic measurements may not be representative of the longer-term environment, and shows how important bioacoustic patterns may be aliased by infrequent sampling strategies. Dusk or nighttime only recording strategies, which continue to be widely used (often with the assumption that these are the times of peak activity), cannot fully capture the non-stationary nature of the marine soundscape.

The snap detection procedure implemented in this study is novel in its use of an envelope correlation procedure that is sensitive to the shape of the arriving pulse. This work also outlines a statistical framework for testing hypotheses related to the call time distribution. This general approach, which is adopted from earthquake triggering studies [46,47], can easily be applied to other call time datasets within bioacoustics.
